# Diving deeper: ways to improve the early identification of young clients with disordered eating in rural areas

**DOI:** 10.1186/2050-2974-3-S1-P4

**Published:** 2015-11-23

**Authors:** Deanne Harris, Lisa Staples, Kathryn Penman, Helen Carter, Leanne Brown

**Affiliations:** 1 Hunter New England Health Local Health District, Newcastle, Australia; 2 Headspace, Australia; 3 University of Newcastle Department of Rural Health Tamworth, Tamworth, Australia

## 

Connections with a local mental health service for young people, headspace, led to collaboration between local health service dietitian's and headspace staff. This partnership enables early detection and treatment of young people with disordered eating in Tamworth NSW.

A project identified the need to enhance staff awareness of nutrition and disordered eating, improved management of young people with disordered eating and streamlined referral processes to local dietitians. Specific education was conducted for headspace, Tamworth staff about food, mood and body image in young people. Staff then attended formal training in identifying and assessing eating disorders.

Staff identified the development of a dietetic service for disordered eating, as a priority for headspace, Tamworth. Standard intake forms were adapted with more detailed questions about intake and attitudes to food and body image. Referral processes to dietitians were improved and referral numbers increased markedly. The demand for eating disorder specific dietetic services led to funding for an on-site dietitian one day per week. Dietitian led monthly clinical case review, staff inservices and young person cooking skills classes have all commenced since the service began.

Improved screening and referral processes have identified the need to ‘dive deeper’ into nutrition for this target group.

